# Ratiometric Strategy for Electrochemical Sensing of Carbaryl Residue in Water and Vegetable Samples

**DOI:** 10.3390/s20051524

**Published:** 2020-03-10

**Authors:** Min Zhang, Zeyuan Zhang, Yanjing Yang, Yi Zhang, Yufei Wang, Xinyi Chen

**Affiliations:** College of Food Science and Engineering, Northwest A&F University, No.22 Xinong Road, Yangling 712100, China; 2016013203@nwafu.edu.cn (Z.Z.); yangyanjing@nwafu.edu.cn (Y.Y.); zyeesccd@nwafu.edu.cn (Y.Z.); wangyufei@nwafu.edu.cn (Y.W.); chenxinyi199997@nwafu.edu.cn (X.C.)

**Keywords:** ratiometric strategy, electrochemical sensor, carbaryl residue, water samples, vegetable samples, carbon cloth

## Abstract

Accurate analysis of pesticide residue in real samples is essential for food safety and environmental protection. However, a traditional electrochemical sensor based on single-signal output is easily affected by background noise, environmental conditions, electrode diversity, and a complex matrix of samples, leading to extremely low accuracy. Hence, in this paper, a ratiometric strategy based on dual-signal output was adopted to build inner correction for sensing of widely-used carbaryl (CBL) for the first time. By comparison, Nile blue A (NB) was selected as reference probe, due to its well-defined peak, few effects on the target peak of CBL, and excellent stability. The effects of a derivatization method, technique mode, and pH were also investigated. Then the performance of the proposed ratiometric sensor was assessed in terms of three aspects including the elimination of system noise, electrode deviation and matrix effect. Compared with traditional single-signal sensor, the ratiometric sensor showed a much better linear correlation coefficient (r > 0.99), reproducibility (RSD < 10%), and limit of detection (LOD = 1.0 μM). The results indicated the introduction of proper reference probe could ensure the interdependence of target and reference signal on the same sensing environment, thus inner correction was fulfilled, which provided a promising tool for accurate analysis.

## 1. Introduction

Carbaryl (CBL, 1-naphthyl-N-methyl carbamate, trade name Sevin) has been extensively used in agriculture due to its broad-spectrum efficacy to control more than 100 species of insect on crops [1]. But the presence of CBL residue in agricultural products and water is a potential hazard to consumers’ health and the environment, because it is a neurotoxin and could cause accumulation in food or water as well as bioconcentration through food chain [2,3]. Therefore, there is a crucial need for monitoring CBL residue in water and minimally processed foods such as vegetables. 

So far, a variety of detection methods have been developed and used for analysis of CBL residue in food and environmental samples [4,5]. The most widely used detection approach is the sensitive and well-established chromatographic technique [6]. However, it is not suitable for on-site analysis because of expensive instruments, complicated operation, and the need for highly toxic organic solvents. Given the above disadvantages, an electrochemical sensor (ECS) becomes promising as an inexpensive, fast, portable tool for tracing pesticide residue on a large scale. Nevertheless, background electric signals of the workstation, variable environmental conditions (e.g., temperature, pH and pollutants), diversity among electrodes, and a complex matrix of samples affect the reproducibility of ECS significantly [7,8]. As it is hard to avoid these variations adopting traditional ECS with single-signal output, the ratiometric ECS (RECS) with dual-signal outputs has emerged to improve reproducibility under the inspiration of the ratiometric fluorescence approach [9,10]. RECS could eliminate various analyte-independent effects via the introduction of an inner reference signal, because the reference probe and target analyte were affected by the same sensing interface [11,12]. With the inner self-rectification calibration, the accuracy and sensitivity in pesticide residue sensing could be further improved. For instance, based on the reference signal of thionine, the RECS for imidacloprid exhibited better reproducibility and lower LOD of 17 nM than 68 nM for single-signal ECS [13]. So far, RECSs have been applied in biomolecules (e.g., dopamine [14,15], uric acid [16], glucose [17,18], DNA [19], thrombin [20], DNA [21,22]), enzymatic activity (e.g., telomerase [23], furin [24]), and mercury ions [25], while less in pesticides. In addition, it is mainly based on a biological approach, for example, the reference probes such as methylene blue (MB) and ferrocene (Fc) were labeled on DNA or aptamers to provide a reference signal [26,27,28]. When it comes to chemical sensors, it is facile to introduce a reference probe by adding in a test electrolyte rather than modification with it on a working electrode [29,30]. Although this approach appears easy to realize, it demands consideration in the stable signal related on the given target analyte, especially in the case of the need for derivitization [31,32]. As the oxidation peak of CBL could be better defined after being hydrolyzed by alkali, the choice of adaptive reference signal become the key process to influence the success of RECS for CBL detection. Once the problem is solved, the strategy is easy and it is efficient to realize in on-site analysis of CBL residue. Hence, in this work, we provide a stable reference signal for CBL. As far as we know, there are few reports on RECS for CBL determination.

Moreover, although ratiometric strategy is thought to eliminate various interferences, there is limited research on the availability of RECS in the elimination of the effect of system noise, diversity among electrodes, and a complex matrix of samples, respectively. With this in mind, we evaluate its availability in CBL determination through the three following aspects: (1) system noise: system noise is well known to be inevitable in various analysis methods such as chromatography, spectroscopy, and electroanalysis. Among these methods, the noise of the electrochemical system is large enough to result in poor reproducibility even in standard solution rather than real samples. Hence, in order to investigate the elimination effect on the noise of the electrochemical system, the most commonly used glassy carbon electrode (GCE) was applied as working electrode in this work, due to its stability compared with other electrodes. (2) Diversity among electrodes: with the development of novel electrodes, more and more new electronalysis methods have been built for the determination of pesticide residue. But the diversity among electrodes formed batch to batch is hard to control, specially with single-signal output. In this work, a type of high conductive, cheap and commercially available textile, known as carbon cloth (CC) [33,34], was utilized as the disposable electrodes to investigate the elimination effect on the diversity among electrodes. In order to further improve the sensitivity, the modification with ionic liquids (IL) which possess high ionic conductivity and wide electrochemical windows was also studied [35,36]. (3) Matrix effect of vegetable samples: because of complex matrix of food sample, electroanalysis technique has not been widely applied in food analysis. Using traditional single-signal ECSs, the matrix effect of food samples was so great as to result in low recoveries. For the sake of higher recoveries, RECSs were speculated to decrease the matrix effect combined with matrix-matched calibration standard method. Above all, ratiometric strategy has been investigated through these three aspects in terms of dynamic range, correlation coefficient, sensitivity, and reproducibility. In this way, an accurate, robust and cost-efficient method has been built for CBL determination in real samples.

## 2. Materials and Methods

### 2.1. Apparatus

Cyclic voltammetry (CV), differential pulse voltammetry (DPV), linear sweep voltammetry (LSV), and square wave voltammetry (SWV) were performed with the computer-controlled CHI 760E electrochemical workstation (Chenhua Instruments Co., Ltd., Shanghai, China). For electrochemical experiments, a 10 mL glass cell with a GCE (3 mm diameter) or CC as the working electrode, platinum wire as the counter electrode, and Ag/AgCl as the reference electrode was used. Prior to electrochemical measurements, the solution was purged with pure nitrogen for 5 min. High-performance liquid chromatography (HPLC) analysis was carried out with an LC-20AT pump and an SPD-M20A detector (Shimadzu, Kyoto, Japan) at 220 nm with a C18 column.

### 2.2. Reagents and Chemicals

**Scheme 1:**
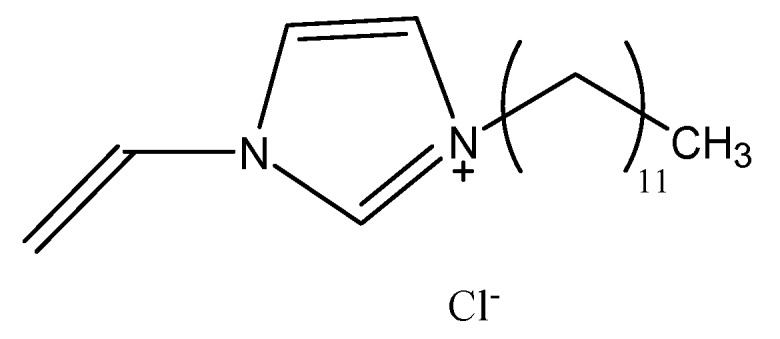
Structures of ion liquid (IL).

CBL, thionine (Th), ferrocene (Fc), methylene blue (MB), Nile blue A (NB) were purchased from Shanghai Aladdin Reagent Co., Ltd. A stock solution of CBL (10^3^ μM) was prepared in ethanol. 0.1 M phosphate-buffered saline (PBS) was used as the solvent-supporting electrolyte system. Conductive CC (HCP330N, 32 cm × 16 cm, 0.32 mm in thickness) was purchased from Hesen Electric Co., Ltd. (Shanghai, China). Ion liquid (IL) was prepared following the methodology of our previous report [37], the structure was shown in Scheme 1. Other reagents used were of analytical reagent grade. 

### 2.3. The Proposed Ratiometric Strategy for Carbaryl (CBL) Determination

**Scheme 2:**
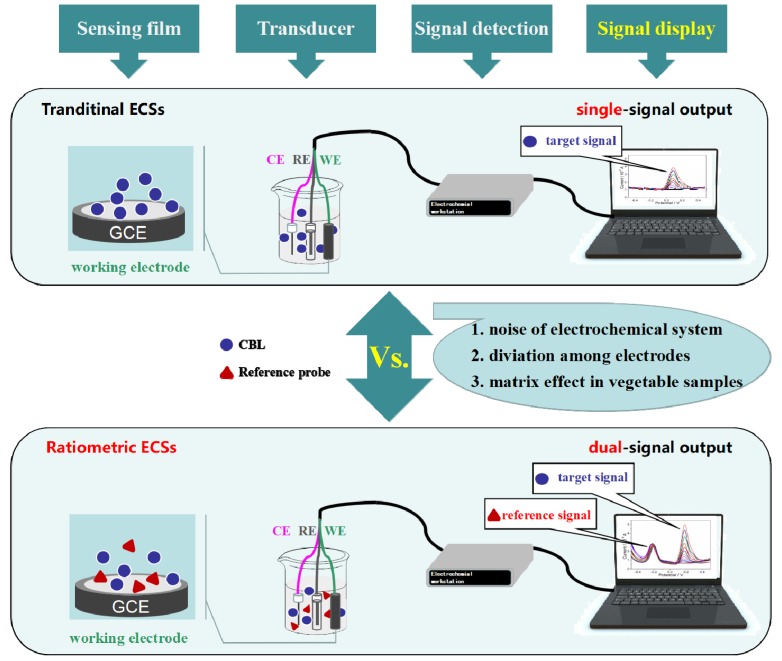
Schematic illustration of electrochemical sensors based on single- and dual-signal sensing strategies.

Electrochemical sensors based on single-signal and dual-signal sensing strategies were illustrated in Scheme 2. Traditional ECS depend on single-signal output, which makes it highly affected by system noise, environmental conditions, electrodes diversity, matrix effect, and so on. Thus, reproducibility is always insufficient to satisfy the requirements of application.

In order to resolve this problem, RECS introduces an inner reference signal to build a self-rectification calibration, because the reference probe and target analyte are affected by the same sensing interface. The reference probe should meet the following conditions: (i) the peaks of probe and CBL are well defined and separated from each other; (ii) the co-existence of the probe could not interfere the signal of CBL; (iii) the probe exhibits a stable signal under the condition that is suitable for CBL determination [38]. In this paper we provide a suitable reference signal for RECS of CBL determination. To illustrate the effect of the reference probe on the availability of ratiometric strategy in electrochemical sensors, the widely used and unmodified GCE was employed at first. In order to further investigate the availability of ratiometric strategy to eliminate the diversity among electrodes and inferences by food matrix, IL/CC was utilized as a disposal electrode and the application experiments was carried out on tomato samples. 

### 2.4. Preparation of Working Electrode

The GCE was polished to a mirror-like surface with 0.05 μm alumina slurry followed by rinsing thoroughly with double distilled water (DDW) before analysis. The CC was firstly cleaned by sonication sequentially in acetone, water, and ethanol for 10 min, respectively. Then, CC was tailored into 5 mm × 10 mm pieces, immersed in 0.25 mg mL^−1^ IL solution for 30 s, and dried in air. The area immersed in solution was 5 mm × 5 mm. 

### 2.5. Electrochemical Analysis 

Before electrochemical analysis, 1.5 mL CBL solution was firstly hydrolyzed with 200 μL 0.2 M NaOH, and 200 μL 0.2 M HCl was added to adjust pH to neutral. Then 100 μL 1000 μM MB solution and 2 mL 0.2 M PBS was added. Finally, the working electrode was accumulated in the mixed solution for 10 min at open circuit potential and all electrochemical analyses were performed from −0.6 to 0.8 V. CV and LSV were carried out at the scan rate of 50 mV s^−1^. SWV and DPV were carried out with the following parameters: increasing potential 0.004 V, amplitude 0.025 V. 

### 2.6. Sample Preparation

Tomatoes and cabbages were purchased from local market, cut into small pieces and homogenized in a blender. 2 g pulp samples were weighed exactly and extracted with 10 mL and 5 mL dichloromethane two times, respectively. Then the lipid substances were removed and dried. 2 mL ethanol and water 1:1 (V/V) was added to redissolve CBL, and filtered using a syringe filter (0.22 μm). Finally, 2 mL 0.2 M PBS and 1 mL DDW were mixed with 1 mL mentioned filtrate before electrochemical analysis. Standard addition method was applied and the results were obtained from an average of three parallel experiments.

### 2.7. High-Performance Liquid Chromatography (HPLC) Measurements

The mobile phase consisted of a mixture of acetonitrile and water 70:30 (V/V), with a flow rate of 0.8 mL min^−1^, and the column temperature was 40 °C. Before HPLC analysis, the extracted samples were filtered using a syringe filter (0.22 μm).

## 3. Results

### 3.1. Ratiometric Strategy on Glassy Carbon Electrode (GCE)

**Figure 1:**
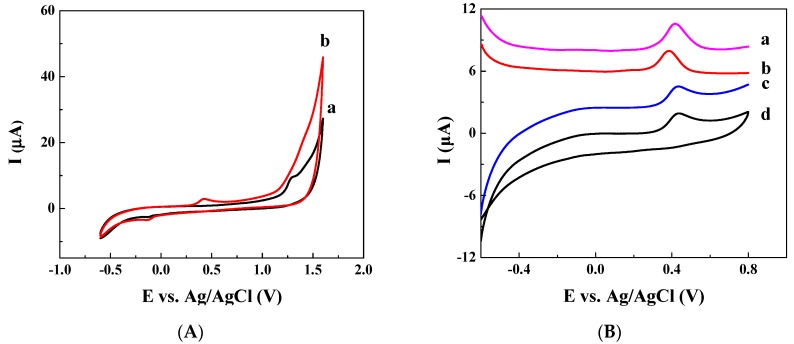
(**A**) Cyclic voltammetry (CV) curves of 25 μM carbaryl (CBL) in 0.1 M phosphate-buffered saline (PBS, pH 7.0) before (a) and after (b) hydrolysis. (**B**) Square wave voltammetry (SWV) (a), differential pulse voltammetry (DPV) (b), linear sweep voltammetry (LSV) (c), and CV (d) curves of 25 μM CBL in 0.1 M PBS (pH 7.0) after hydrolysis.

To investigate the effect of ratiometric strategy on the electrochemical sensor system, the most common GCE was selected as the working electrode. The electrochemical behavior of CBL molecules directly at bare GCE was shown in Figure 1. It exhibited a undefined peak at about 1.1 V, which is difficult for quantifying determination. In order to resolve this problem, derivation strategy was applied based on the fact that CBL can be hydrolyzed to high electroactive 1-naphtol in alkaline medium (Equation (1)). Then, OH- extracts -H from 1-naphthol, a well-defined oxidation peak appearing at 0.4 V, a much lower potential (Equation (2)). This is beneficial for sensitive determination of CBL. The alkalized CBL was expressed as a-CBL in the following text. For the sake of high signal response, different electrochemical techniques including LSV, DPV, SWV and CV were compared in Figure 1B. It is obvious that the SWV curve exhibited a much better defined peak and higher peak current. Accordingly, the SWV technique was utilized in the following electroanalysis.

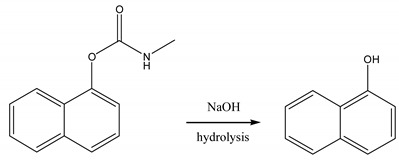
(1)

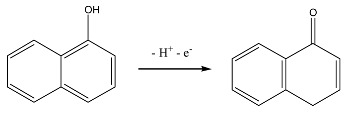
(2)

**Figure 2:**
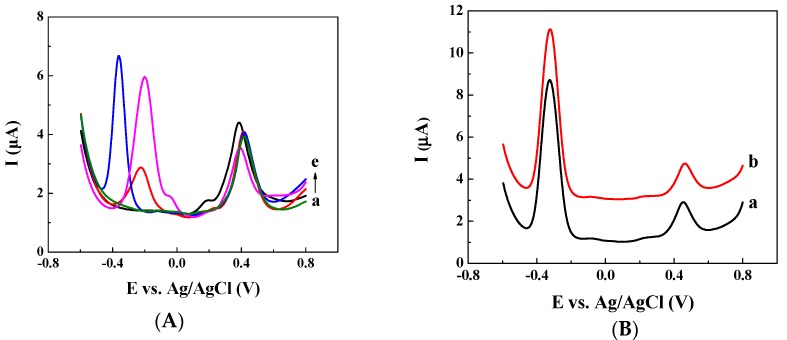
(**A**) SWV curves of 25 μM CBL in 0.1 M PBS (pH 7.0) together with various reference probes. (a) without probes; (b) ferrocene (Fc); (c) methylene blue (MB); (d) thionine (Th); (e) Nile blue (NB). (**B**) SWV curves of 25 μM CBL and 5 μM NB in different adding order: (a) CBL, NaOH, NB; (b) CBL, NB, NaOH.

The reference signal is an essential element in RECS [39,40]. Hence, several common probes with electrochemical activity have been investigated for the fabrication of CBL-RECSs. The electrochemical behaviour of the four probes is shown in Figure 2A. It can be seen that the peak potential of Fc is too near with the derivation product of CBL (curve b) and there was also a weak peak of MB next to a-CBL (curve c), which means Fc and MB were not able to separate well with a-CBL. At the same time, the peaks of Th (curve d) and NB (curve e) were well defined so that they are more suitable to be reference probes for electrochemical determination of CBL in this regard. In addition, compared with a-CBL without any probes (curve a), the introduction of Th obviously reduced the peak current of a-CBL, making it unsuitable for CBL determination. Till now, NB seemed to be the most probable reference probe for CBL. In order to verify the conjecture, stability experiments were also carried out. The RSD of peak current ratio between a-CBL and NB was calculated to be only 2.5%, when the mixture was tested for 5 times under the same conditions every ten minutes. Besides, the adding sequence of reference probe and alkali were also investigated. As shown in Figure 2B, the peaks of a-CBL and NB seemed to be the same no matter adding NB before NaOH or after NaOH, which further indicated the stability of NB in the condition of CBL determination. To sum up, NB was selected as the reference probe for ratiometric electrochemically sensing CBL, due to the well-defined oxidation peak, few effect on the peak of CBL, and the excellent stability which was accordance with previous report [41]. These three conditions of reference probes are essential to meet the requirements of RECS mentioned above in Section 3.1. 

**Figure 3:**
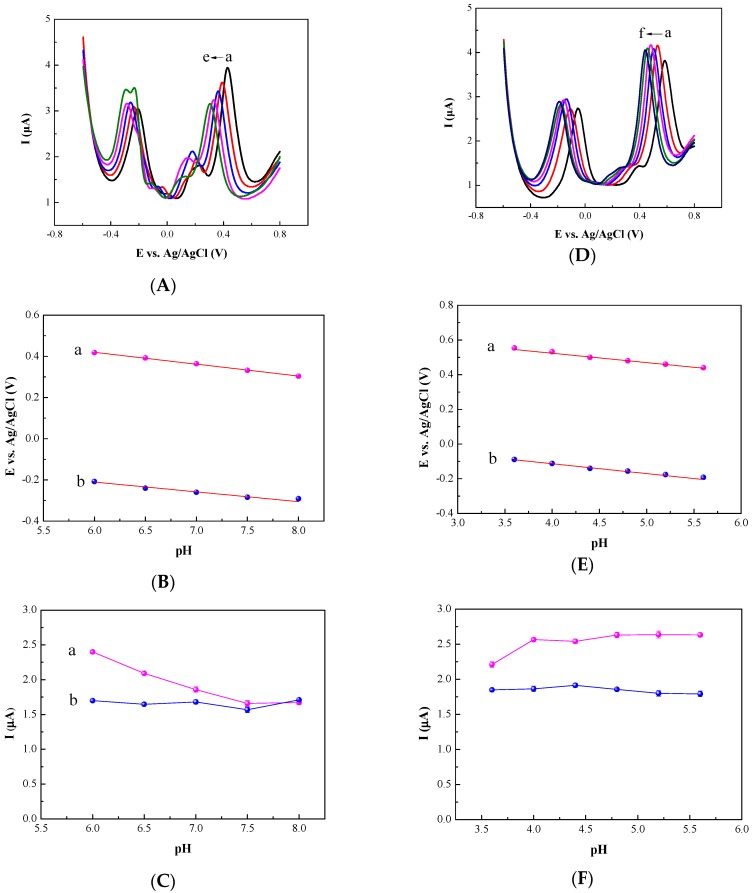
(**A**) SWV curves of 75 μM CBL and 5 μM NB in PBS with various pH value: (a) 6.0; (b) 6.5; (c) 7.0; (d) 7.5; (e) 8.0; (**B**) plots of peak potential and pH value in PBS (a) CBL, (b) NB. (**C**) Plots of peak current and pH value (a) CBL, (b) NB. (**D**) SWV curves of 75 μM CBL and 5 μM NB in acetate buffer solution (ABS) with various pH value: (a) 3.6; (b) 4.0; (c) 4.4; (d) 4.8; (e) 5.2; (f); 5.6. (**E**) Plots of peak potential and pH value in PBS (a) CBL, (b) NB. (**F**) Plots of peak current and pH value in ABS (a) CBL, (b) NB.

The pH value is an important parameter in electroanalysis, so the electrochemical response of a-CBL and a-NB in PBS of different pH value from 6.0 to 8.0 were investigated by SWV. As shown in Figure 3, the peak potential of NB and a-CBL shifted to positive potentials for decreasing pH values. The relationships were linear with the slope of 54 and 48 mV/pH, respectively (r > 0.99), which implies that both of a-NB and a-CBL oxidation follows the Nernst equation requiring identical number of protons and electrons. Besides, the peak current of a-CBL increases with the decrease of pH values, because the 1-naphthol molecules can be protonated at low pH. In order to investigate the effect of lower pH value, the use of acetate buffer solution (ABS) with a pH value from 3.6 to 5.6 was also studied. As shown in Figure 3, the trend of peak potentials changed the same way as in PBS, and the peak current decreased when the pH value decreased to 3.6. In addition, ABS is more volatile than PBS, which may lead to instability of detection. Accordingly, 0.1 M pH 6.0 PBS was selected as the appropriate supporting electrolyte in RECSs for CBL determination. 

**Figure 4:**
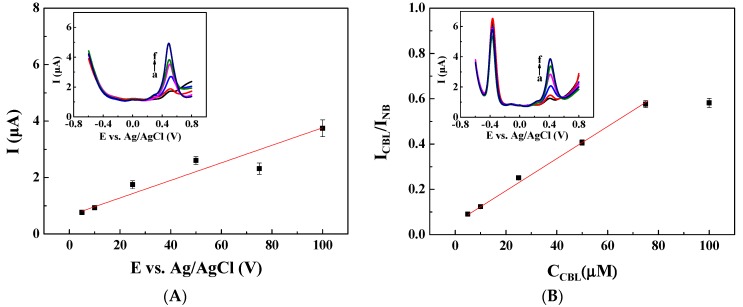
(**A**) Calibration curves of CBL using single-signal strategy on bare GCE; (**B**) Calibration curves of CBL using dual-signal strategy on bare GCE. Inset: SWV curves of various concentration of CBL. Error bars: SD; n = 3.

Then the analytical performance were investigated in terms of the ratiometric strategy on GCE. As shown in Figure 4A, the relationship between the current ratio and CBL concentration was highly linear between 5 and 75 μM and described as i_CBL_/i_MB_ = 0.007 C_CBL_ + 0.061 with r of 0.999. The LOD was calculated to be 1.0 μM at a signal-to-noise ratio of 3. Compared with the single-signal ECSs (Figure 4B), the equation of the peak current and CBL concentration was i_CBL_ = 0.029 C_CBL_ + 0.780 with r of 0.947, which is lower than RECSs. Moreover, the reproducibility was also investigated through series of 5 prepared electrodes under the same conditions at short time interval with RSD of 2.8% for RECSs, while 7.2% for traditional ECSs. It is clear that ratiometric strategy is beneficial to eliminate the background noise of electrochemical workstation, giving rise to higher accuracy and reproducibility.

### 3.2. Ratiometric Strategy on Carbon Cloth (CC) Electrode

**Figure 5:**
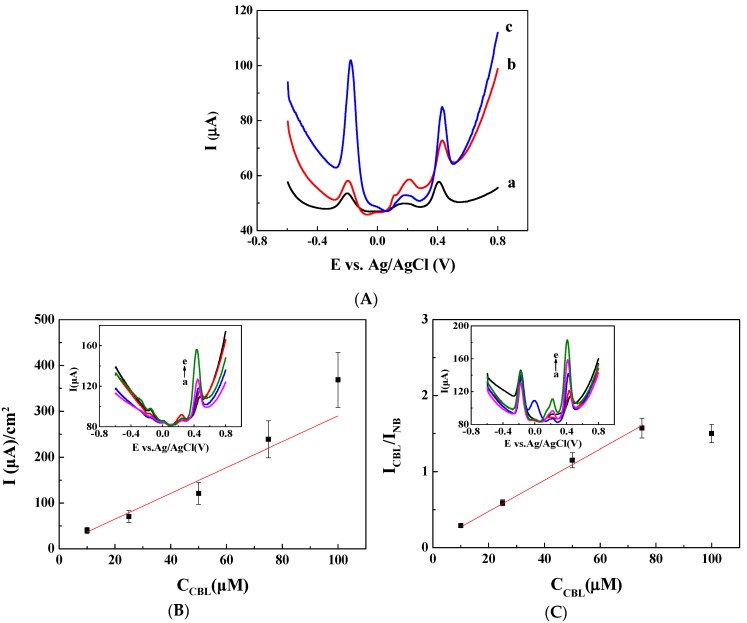
(**A**) SWV curves of 50 μM CBL and 1 μM NB at glassy carbon electrode (GCE) (a), carbon cloth (CC) (b), and IL/CC (c); (**B**) calibration curves of CBL using single-signal on IL/CC. (**C**) Calibration curves of CBL using dual-signal on IL/CC. Inset: SWV curves of various concentration of CBL. Error bars: standard deviation (SD); n = 3.

The advantages of ratiometric strategy in ECS reflected not only on the background noise of the electrochemical system, but also the diversity among the electrodes. In order to verify it, CC was selected as the working electrode, because the size immersed in electrolyte solution is hard to control and measure, which greatly affects the accuracy of CBL determination using single-signal ECS. Besides, high conductive CC will improve the sensitivity of CBL determination sharply. In order to further improve the signal response, IL was utilized to modify CC. As shown in Figure 5A, the peak currents of a-CBL and NB were greatly enhanced after the simple soaking of CC in IL solution. The concentrations of IL were also investigated, and 0.25 mg mL^−1^ was the optimized value. 

Then the linear calibration range, sensitivity, and reproducibility were investigated to evaluate the proposed strategy at the IL/CC electrode. As shown in Figure 5B,C, the peak currents of a-CBL increased with increasing concentration of CBL while the peak currents of NB were not changed. The current ratio of a-CBL and NB was linear with the concentration of CBL ranging from 10 to 75 μM and the regression equation was: i_CBL_/i_MB_ = 0.021 C_CBL_ + 0.112 (r = 0.999). The LOD was calculated to be 1.4 μM at a signal-to-noise ratio of 3. At the same time, i_CBL_/cm^2^ = 3.627 C_CBL_ - 21.161 (r = 0.980) was also fitted for a single-signal IL/CC sensor. In addition, the electrode-to-electrode reproducibility (RSD, n = 5) for CBL (25 μM) was determined as 6.8%, much lower than the 21.0% of single-signal ECSs. The results indicated the ratiometric strategy was available at novel CC electrode except for GCE. 

The performance of this sensor was compared with other sensors for CBL, which are listed in Table 1. The detection limit and linear calibration range of the proposed sensor are comparable with some single-electronic sensors and not better than some others. Nevertheless, the process of fabrication is most simple of all, which is easy to realize. Furthermore, the proposed ratiometric strategy could be compatible with various modified electrodes to further improve the detection performance through a simple process and the requirement of a common reagent.

### 3.3. Ratiometric Strategy in Vegetable Samples

**Figure 6:**
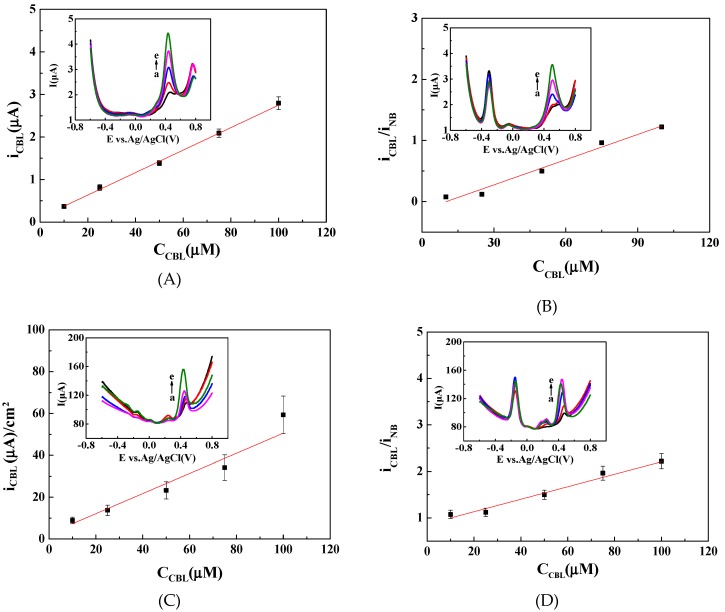
(**A**) Calibration curves of CBL using single-signal strategy on bare GCE in tomato samples. (**B**) Calibration curves of CBL using dual-signal strategy on bare GCE in tomato samples. (**C**) Calibration curves of CBL using single-signal strategy on IL/CC in tomato samples. (**D**) Calibration curves of CBL using dual-signal strategy on IL/CC in tomato samples. Isnet: SWV curves of various concentration of CBL. Error bars: SD; n = 3.

The application in real samples is always the challenge in research area of ECS because of complex matrix and tedious pre-treatment process. As a potential technique for a rapid test of pesticide residue in agriculture products, the pre-treatment of ECS should be as simple as possible. Based on the previous research, various extraction solvents have been applied to extract trace CBL from various samples. Accordingly, the extraction experiment in vegetable samples was carried out firstly. The extraction rates of extraction solvents including dichloromethane, *n*-hexanem, acetonitrile, and methanol were calculated to be 94.05% ± 2.92%, 90.86% ± 1.98%, 72.91% ± 2.55%, and 65.06% ± 4.11%, respectively. Hence, dichloromethane was chosen to be the extraction solvent. After cleaning with the liquid-liquid extraction process, the peak current of CBL decreased, while the peak current of impurity slightly decrease. This means the clean-up process is unnecessary and the simple extraction with dichloromethane is sufficient for accurate CBL sensing. Using this simple pretreatment process, the calibration of CBL in tomato samples were carried out in Figure 6. On GCE, the regression equation was i_CBL_/i_MB_ = 0.014 C_CBL_ − 0.140 (r = 0.999) for RECSs, while i_CBL_ = 0.027 C_CBL_ + 0.105 (r = 0.990) for ECSs. On IL/CC, the regression equation was i_CBL_/i_MB_ = 0.014 C_CBL_ + 0.860 (r = 0.990) for RECSs, while i_CBL_/cm^2^ = 0.533 C_CBL_ + 0.155 (r = 0.970) for ECSs. The results indicated that the ratiometric strategy slightly improves the detection accuracy. Moreover, as shown in Table 2, the recovery rates in tomato and cabbage samples were between 80% and 95%, which is much better than other reported sensors [3]. Hence, it was speculated that the matrix effect was the major cause of inaccurate CBL detection when the matrix is complex, and the matrix-matching standard solution method played a more important role compared with ratiometric strategy in this case. In a combination of the two strategy, the recoveries could be improved and comparable with the HPLC method.

### 3.4. Comparison between Ratiometric Electrochemical Sensor (RECS) and Electrochemical Sensor (ECS)

In order to make the advantages of the ratiometric strategy clear, a comparison between RECS and ECS is shown in Table 3. When the matrix is water, the linear correlation coefficient (r) and relative standard deviation (RSD) of RECS were obviously higher than ECS, indicating the ratiometric strategy is available for the accurate determination of CBL in water samples. At the same time, there were no significant difference between that of RECS and ECS when the matrix is tomato sample, which means the matrix-matching standard solution method is more effective in the elimination of matrix effect on CBL determination. In conclusion, the ratiometric strategy is highly effective at eliminating the effect of system noise and electrodes deviation on CBL determination, leading to accurate sensing of CBL in water samples. 

## 4. Conclusions

In this work, a ratiometric strategy has been successfully applied in the electrochemical determination of CBL residue by the direct addition of a proper reference probe after rapid hydrolysis. Taking consideration of the well-defined peak, few effects on the target peak, and excellent stability, Nile blue A was selected as the probe to provide a reference signal for ratiometric electrochemical sensing of CBL. The introduction of a proper probe could ensure the interdependence of the target signal and reference signal on the same sensing environment including system noise, electrode deviation, and co-existing molecules adsorbed on the surface of the electrode. Thus, their ratio value could eliminate the similar interference on the analytical signal, leading to higher accuracy. Based on this dual-signal model, the proposed ratiometric sensor has been proved to be available to reduce the effect of system noise and electrode deviation on CBL determination compared with the traditional single-signal sensor. Therefore, inner correction was fulfilled, providing a promising tool for accurate analysis of CBL residue in practical applications.

## Figures and Tables

**Table 1:** Comparison of analytical parameters on various electrodes.

Electrode	Linear Range	Limit of Detection (LOD, μM)	Ref.
CB-NPs electrode	25~125	12	[42]
AChE/PANI/MWCNTs/GCE	9.9~49.6	1.4	[43]
LSX zeolite/CPE	1~100	0.3	[44]
BDDGR	1~6	0.07	[45]
CoO/rGO/GCE	0.2~70	0.04	[46]
GO-IL/GCE	0.10~12.0	0.02	[47]
Bare GCE	5~75	1.0	This work
IL/CC	10~75	1.4	This work

Notes: CB-NPs—carbon black nanoparticles; AChE/PANI/MWCNTs/GCE—acetylinesterase/polyaniline/multi-walled carbon nanotubes/glassy carbon electrode; LSX zeolite/CPE—low silica X zeolite modified carbon paste electrode; BDDGR—graphene modified boron-doped diamond electrode; CoO/rGO—cobalt oxide decorated reduced graphene oxide; GO-IL—graphene oxide-ionic liquid composite.

**Table 2:** Determination results for CBL in real samples.

Sample	Amount Added (μM)	Amount Recovered (μM)	Recovery Rate (%)	Content Determined by SWV (mg kg^−1^) ± SD	Content Determined by HPLC (mg kg^−1^) ± SD
Tomato	10.00	8.11	81.10	1.63 ± 0.08	1.77 ± 0.05
	25.00	21.22	84.88	4.27 ± 0.11	4.59 ± 0.09
	50.00	43.94	87.88	8.84 ± 0.29	9.11 ± 0.12
Cabbage	10.00	8.72	87.20	1.75 ± 0.15	1.81 ± 0.04
	25.00	23.19	92.76	4.66 ± 0.27	4.44 ± 0.15
	50.00	42.97	85.94	8.65 ± 0.14	8.99 ± 0.05

**Table 3:** Comparison between ratiometric electrochemical sensor (RECS) and electrochemical sensor (ECS).

Interference Factor	Electrodes	Matrix	RECS			ECS		
			Linear Relationship	r	RSD (%)	Linear Relationship	r	RSD (%)
System noise	GCE	Water	i_CBL_/i_MB_ = 0.007C_CBL_ + 0.061	0.999	2.8	i_CBL_ = 0.029 C_CBL_ + 0.780	0.947	7.2
Electrodes deviation	IL/CC	Water	i_CBL_/i_MB_ = 0.021C_CBL_ + 0.112	0.999	6.8	i_CBL_/cm^2^ = 3.627 C_CBL_ − 21.161	0.980	21.0
Matrix effect in vegetable samples	GCE	Tomato sample	i_CBL_/i_MB_ = 0.014C_CBL_ − 0.140	0.999	5.8	i_CBL_ = 0.027 C_CBL_ + 0.105	0.990	5.9
	IL/CC	Tomato sample	i_CBL_/i_MB_ = 0.014C_CBL_ + 0.860	0.990	9.2	i_CBL_/cm^2^ = 0.533 C_CBL_ + 0.155	0.970	17.0

## References

[1] Zhang C., Ma G.P., Fang G.Z., Zhang Y., Wang S. (2008). Development of a capillary electrophoresis-based immunoassay with laser-induced fluorescence for the detection of carbaryl in rice samples. J. Agric. Food Chem..

[2] Dong T.T., Sun J.W., Liu B., Zhang Y., Song Y., Wang S. (2010). Development of a sensitivity-improved immunoassay for the determination of carbaryl in food samples. J. Sci. Food Agric..

[3] Zhang C., Cui H.Y., Cai J.R., Duan Y.Q., Liu Y. (2015). Development of fluorescence sensing material based on CdSe/ZnS quantum dots and molecularly imprinted polymer for the detection of carbaryl in rice and Chinese cabbage. J. Agric. Food Chem..

[4] Alsammarraie F.K., Lin M.S. (2017). Using standing gold nanorod arrays as surface-enhanced raman spectroscopy (SERS) substrates for detection of carbaryl residues in fruit juice and milk. J. Agric. Food Chem..

[5] Chen W., Liu Y.Y., Zhang Y., Fang J.H., Xu P.C., Xu J.Q., Li X.X., Liu C.C., Wen W.J. (2017). Highly effective and specific way for trace analysis of carbaryl insecticides based on Au_42_Rh_58_ alloy nanocrystals. J. Mater. Chem. A..

[6] Sun B., Wang C.P., Wang Q., Chen L., Dang X.P., Huang J.L., Chen H.X. (2017). Preparation of acryloyl beta-cyclodextrin organic polymer monolithic column and its application in solid-phase microextraction and HPLC analysis for carbofuran and carbaryl in rice. Food Anal. Method.

[7] Sahoo D., Mandal A., Mitra T., Chakraborty K., Bardhan M., Dagupta A.K. (2018). Nanosensing of pesticides by zinc oxide quantum dot: An optical and electrochemical approach for the detection of pesticides in water. J. Agric. Food Chem..

[8] Abdalhai M.H., Fernandes A.M., Xia X., Musa A., Ji J., Sun X. (2015). Electrochemical genosensor to detect pathogenic bacteria (*Escherichia coli* O157:H7) as applied in real food samples (fresh beef) to improve food safety and quality control. J. Agric. Food Chem..

[9] Jiang X.Q., Yu Y., Chen J.W., Zhao M.K., Chen H., Song X.Z., Matzuk A.J., Carroll S.L., Tan X., Sizovs A. (2015). Quantitative imaging of glutathione in live cells using a reversible reaction-based ratiometric fluorescent probe. ACS Chem. Bio..

[10] Komatsu K., Urano Y., Kojima H., Nagano T. (2007). Development of an iminocoumarin-based zinc sensor suitable or ratiometric fluorescence imaging of neuronal zinc. JACS.

[11] Xiong E., Li Z.Z., Zhang X.H., Zhou J.W., Yan X.X., Liu Y.Q., Chen J.H. (2017). A triple-helix molecular switch electrochemical ratiometric biosensor for ultrasensitive detection of nucleic acids. Anal. Chem..

[12] Manibalan K., Mani V., Chang P.C., Huang C.H., Huang S.T., Marchlewicz K., Neethirajan S. (2017). Electrochemical latent redox ratiometric probes for real-time tracking and quantification of endogenous hydrogen sulfide production in living cells. Biosens. Bioelectron..

[13] Li X., Kan X. (2018). A ratiometric strategy-based electrochemical sensing interface for the sensitive and reliable detection of imidacloprid. Analyst.

[14] Yang J., Hu Y., Li Y. (2019). Molecularly imprinted polymer-decorated signal on-off ratiometric electrochemical sensor for selective and robust dopamine detection. Biosens. Bioelectron..

[15] Jin H., Zhao C., Gui R., Gao X., Wang Z. (2018). Reduced graphene oxide/nile blue/gold nanoparticles complex modified glassy carbon electrode used as a sensitive and label-free aptasensor for ratiometric electrochemical sensing of dopamine. Anal. Chim. Acta.

[16] Gao X., Gui R., Xu K.Q., Guo H., Jin H., Wang Z. (2018). A bimetallic nanoparticle/graphene oxide/thionine composite-modified glassy carbon electrode used as a facile ratiometric electrochemical sensor for sensitive uric acid determination. New J. Chem..

[17] Xu M., Wang L., Xie Y., Song Y., Wang L. (2019). Ratiometric electrochemical sensing and biosensing based on multiple redox-active state COF_DHTA-TTA_. Sens. Actuators B.

[18] Gong C., Shen Y., Song Y., Wang L. (2017). On-off ratiometric electrochemical biosensor for accurate detection of glucose. Electrochim. Acta.

[19] Wang L., Gong C., Shen Y., Ye W., Xu M., Song Y. (2017). A novel ratiometric electrochemical biosensor for sensitive detection of ascorbic acid. Sens. Actuators B.

[20] Wang L., Ma R., Jiang L., Jia L., Jia W., Wang H. (2017). A novel “signal-on/off” sensing platform for selective detection of thrombin based on target-induced ratiometric electrochemical biosensing and biobar-corded nanoprobe amplification strategy. Biosens. Bioelectron..

[21] Ma R.N., Wang L.L., Wang H.F., Jia L.P., Zhang W., Shang L., Xue Q.W., Jia W.L., Liu Q.Y., Wang H.S. (2018). Highly sensitive ratiometric electrochemical DNA biosensor based on homogeneous exonuclease Ⅲ-assisted target recycling amplification and one-step triggered dual-signal output. Sens. Actuators B.

[22] Yang T., Yu R., Liu S., Qiu Z., Luo S., Li W., Jiao K. (2018). A ratiometric electrochemical deoxyribonucleic acid sensing strategy based on self-signal of highly stable reduced graphene oxide-flavin mononucleotide aqeous dispersion modified nanointerface. Sens. Actuators B.

[23] Dong P., Zhu L., Huang J., Ren J., Lei J. (2019). Electrocatalysis of cerium metal-organic frameworks for ratiometric electrochemical detection of telomerase activity. Biosens. Bioelectron..

[24] Yao D., Zhao W., Zhang L., Tian Y. (2017). A ratiometric electrochemical strategy for sensitive determinaiton of furin activity based on dual signal amplification and antifouling nanosurfaces. Analyst.

[25] Xiong E., Wu L., Zhou J., Yu P., Zhang X., Chen J. (2015). A ratiometric electrochemical biosensor for sensitive detection of Hg^2+^ based on thymine-Hg^2+^-thymine structure. Anal. Chim. Acta.

[26] Li Y., Chang Y., Ma J., Wu Z., Yuan R., Chai Y. (2019). Programming a target-initiated biofunctional DNAzyme nanodevice for sensitive ratiometric electrochemical biosensing. Anal. Chem..

[27] Lin Y., Jia J., Yang R., Chen D., Wang J., Luo F., Guo L., Qiu B., Lin Z. (2019). Ratiometric immunosensor for GP73 detection based on the ratios of electrochemiluminescence and electrochemical signal using DNA tetrahedral nanostructure as the carrier of stable reference signal. Anal. Chem..

[28] Ge L., Wang W., Li F. (2017). Electro-grafted electrode with graphene-oxide-like DNA affinity for ratiometric homogenous electrochemical biosensing of microRNA. Anal. Chem..

[29] Li S., Tian Y. (2018). An electrochemical biosensor with dual signal outputs for ratiometric monitoring the levels of H_2_O_2_ and pH in the microdialysates from a rat brain. Electroanalysis.

[30] Yu Y., Wang P., Zhu X., Peng Q., Zhou Y., Yin T., Liang Y., Yin X. (2018). Combined determination of copper ions and β-amploid peptide by a single ratiometric electrochemical biosensor. Analyst.

[31] Yu J.B., Jin H., Gui R.J., Wang Z.H., Ge F. (2017). A general strategy to facilely design ratiometric electrochemical sensors in electrolyte solution by directly using a bare electrode for dual-signal sensing of analytes. Talanta.

[32] Zhao C.Q., Jin H., Gui R.J., Wang Z.H. (2017). Facile fabrication of dual-ratiometric electrochemical sensors based on a bare electrode for dual-signal sensing of analytes in electrolyte solution. Sens. Actuators B.

[33] Meng S.J., Hong Y., Dai Z.Y., Huang W., Dong X.C. (2017). Simultaneous detection of dihydroxybenzene isomers with ZnO nanorod/carbon cloth electrodes. ACS Appl. Mater. Inter..

[34] Wang X.D., Zheng Y.Y., Yuan J.H., Shen J.F., Hu J.G., Wang A.J., Wu L.J., Niu L. (2017). Three-dimensional NiCo layered double hydroxide nanosheets array on carbon cloth, facile preparation and its application in highly sensitive enzymeless glucose detection. Electrochim. Acta..

[35] Luan F., Zhang S., Chen D.D., Zheng K., Zhuang X.M. (2018). CoS_2_-decorated ionic iquid-functionalized graphene as a novel hydrazine electrochemical sensor. Talanta.

[36] Chaiyo S., Mehmeti E., Siangproh W., Hoang T.L., Nguyen H.P., Chailapakul O., Kalcher K. (2018). Non-enzymatic electrochemical detection of glucose with a disposable paper-based sensor using a cobalt phthalocyanine-ionic liquid-graphene composite. Biosens. Bioelectron..

[37] Du C.B., Hu X.L., Guan P., Guo L.X., Qian L.W., Song R.Y., Wang C.L. (2015). Water-compatible surface-imprinted microspheres for high adsorption and selective recognition of peptide drug from aqueous media. J. Mater. Chem. B.

[38] Yang T., Yu R.Z., Yan Y.H., Zeng H., Luo S.Z., Liu N.Z., Morrin A., Luo X.L., Li W.H. (2018). A review of ratiomentric electrochemical sensors: From design schemes to future prospects. Sens. Actuators B.

[39] Spring S.A., Goggins S., Frost C.G. (2017). Ratiometric electrochemical detection of β-galactosidase. Org. Biomol. Chem..

[40] Goggins S., Naz C., Marsh B.J., Frost C.G. (2015). Ratiometric electrochemical detection of alkaline phosphatase. Chem. Commun..

[41] Gao X., Gui R., Guo H., Wang Z., Liu Q. (2019). Creatinine-induced specific signal responses and enzymeless ratiometric electrochemical detection based on copper nanoparticles electrodeposited on reduced graphene oxide-based hybrids. Sens. Actuators B.

[42] Della Pelle F., Del Carlo M., Sergi M., Compagnone D., Escarpa A. (2016). Press-transferred carbon black nanoparticles on board of microfluidic chips for rapid and sensitive amperometric determination of pheyl carbamate pesticides in environmental samples. Microchim. Acta..

[43] Cesarino I., Moraes F.C., Lanza M.R.V., Machado S.A.S. (2012). Electrochemical detection of carbamate pesticides in fruit and vegetables with a biosensor based on acetylcholinesterase immobilised on a composite of polyaniline-carbon nanotubes. Food Chem..

[44] Salih F.E., Achiou B., Ouammou M., Bennazha J., Ouarzane A., Younssi S.A., El Rhazi M. (2017). Electrochemical sensor based on low silica X zeolite modified carbon paste for carbaryl determination. J. Adv. Res..

[45] Pop A., Manea F., Flueras A., Schoonman J. (2017). Simultaneous voltammetric detection of carbaryl and paraquat pesticides on graphene-modified boron-doped diamond electrode. Sensors.

[46] Wang M.Y., Huang J.R., Wang M., Zhang D.E., Chen J. (2014). Electrochemical nonezymatic sensor based on CoO decorated reduced graphene oxide for the simultaneous determination of carbofuran and carbaryl in fruits and vegetables. Food Chem..

[47] Liu B.Z., Xiao B., Cui L.Q. (2015). Electrochemical analysis of carbaryl in fruit samples on graphene oxide-ionic liquid composite modified electrode. J. Food Compos. Anal..

